# Altered FoxO1 and PPARγ interaction in age-related ER stress-induced hepatic steatosis

**DOI:** 10.18632/aging.102042

**Published:** 2019-06-25

**Authors:** Dae Hyun Kim, Sugyeong Ha, Yeon Ja Choi, H. Henry Dong, Byung Pal Yu, Hae Young Chung

**Affiliations:** 1 Department of Pharmacy, College of Pharmacy, Pusan National University, Busan 46241, Korea; 2 Department of Biopharmaceutical Engineering, Division of Chemistry and Biotechnology, Dongguk University, Gyeongju 38066, Korea; 3 Department of Pediatrics, Children’s Hospital of Pittsburgh of UPMC, School of Medicine, University of Pittsburgh, Pittsburgh, PA 15224, USA; 4 Department of Physiology, The University of Texas Health Science Center at San Antonio, San Antonio, TX 78229, USA

**Keywords:** aging process, FoxO1, PPARγ, ER stress, lipid accumulation

## Abstract

Chronic kidney disease (CKD) is one of the most powerful predictors of premature cardiovascular disease (CVD), with heightened susceptibility to vascular intimal and medial calcification associated with a high cardiovascular mortality. Abnormal mineral metabolism of calcium (Ca) and phosphate (P) and underlying (dys)regulated hormonal control in CKD-mineral and bone disorder (MBD) is often accompanied by bone loss and increased vascular calcification (VC). While VC is known to be a multifactorial process and a major risk factor for CVD, the view of primary triggers and molecular mechanisms complexity has been shifting with novel scientific knowledge over the last years. In this review we highlight the importance of calcium-phosphate (CaP) mineral crystals in VC with an integrated view over the complexity of CKD, while discuss past and recent literature aiming to highlight novel horizons on this major health burden. Exacerbated VC in CKD patients might result from several interconnected mechanisms involving abnormal mineral metabolism, dysregulation of endogenous calcification inhibitors and inflammatory pathways, which function in a feedback loop driving disease progression and cardiovascular outcomes. We propose that novel approaches targeting simultaneously VC and inflammation might represent valuable new prognostic tools and targets for therapeutics and management of cardiovascular risk in the CKD population.

## INTRODUCTION

Forkhead box O1 (FoxO1) proteins are mammalian evolutionarily conserved transcription factors and include FoxO1, FoxO3a, FoxO4, and FoxO6 [1]. FoxOs are regulated by various nutritional or molecular factors. Phosphorylation is a key-mechanism of FoxOs regulation. For instance, Akt-mediated phosphorylation inactivates FoxO by inducing its translocation from the nucleus to the cytoplasm [2–4]. In addition, it has been reported that elevated intracellular reactive oxygen species (ROS) and fatty acids (e.g., palmitate) activate FoxOs via a distinct mechanism involving JNK signaling [5, 6]. Although the role of FoxO1 in the control of gluconeogenesis-related gene expression is widely recognized, its involvement in the regulation of hepatic lipid metabolism remains uncertain. In some studies, it was reported [7] that in the liver, the expression of a constitutively active version of FoxO1 induced lipogenic sterol regulatory element binding protein 1c (SREBP-1c) gene expression and hepatic triglyceride (TG) accumulation, although others have not replicated these findings. One study in mice reported that FoxO1 is necessary and sufficient to promote the production of hepatic very-low-density lipoprotein-associated TG (VLDL-TG) and induce hypertriglyceridemia via the regulation of microsomal TG transfer protein (MTP) [8]. Interestingly, FoxO1 was shown to repress adipocyte differentiation via peroxisome proliferator-activated receptors γ (PPARγ) [9] and several other lines of evidence demonstrate that FoxO1 binds to the PPARγ promoter to repress its transcription [10]. Further studies are necessary to characterize the role of FoxO1 in the control of hepatic lipid metabolism during aging.

PPARs belong to the nuclear hormone receptor superfamily, comprising ligand-modulated transcription factors. PPARs heterodimerizes with retinoid X receptors (RXRs) and binds to PPAR response elements (PPRE) in the promoter region of specific target genes, regulating their transcription. Currently, three PPAR subtypes, PPARα, PPARβ, and PPARγ have been identified. Among them, PPARγ promotes adipogenesis, controls lipid accumulation in adipocytes, and regulates the expression of adipocyte-secreted proteins and adipocytokines (leptin and adiponectin) to reduce lipotoxicity [11] and hepatic lipid metabolism [12, 13]. Kim et al., [14] reported that in diabetic db/db mice, hepatic FoxO6 significantly induced hepatic PPARγ expression in insulin resistant liver, and consequently induced hepatic lipogenesis as well as increased hepatic fat content. However, the molecular interaction between FoxO1 and PPARγ in aging-related lipid accumulation by endoplasmic reticulum (ER) stress has not been reported.

Aging represents the accumulation of deleterious changes in a organism over time, leading to physical and functional deteriorations. Age-related diseases include diabetes, cancer, arthritis, dementia, vascular diseases, obesity, and metabolic syndrome [15]. The elderly population exhibits an increased incidence of diabetes and obesity, which are causally associated with insulin resistance [16]. Insulin resistance is a pathophysiological condition in which cells fail to respond to normal insulin signals to store glucose in the tissues. As a result, hyperglycemia and hyperinsulinemia occur because of the reduced glucose uptake from tissues in response to insulin and the consequent increase in insulin secretion by pancreatic beta cells, in the attempt to control glucose homeostasis. In addition, diabetics overproduce glucose and TG, contributing to the twin abnormalities of this disease, i.e., hyperglycemia and hypertriglyceridemia [17]. Less clear is how these insulin actions are mediated, and why they are inextricably linked to the pathogenesis of insulin resistance that is the forerunner of aging-related type-2 diabetes. Aging-related increases in ER stress also play a big role in insulin resistance [18–22]. However, the molecular mechanism by which ER stress can lead to aging-related hepatic steatosis needs to be further investigated.

The ER is an organelle that known to regulate cellular calcium storage, protein synthesis, and protein folding. An accumulation of unfolded or misfolded proteins in the lumen of the ER induces ER stress and unfolded protein response (UPR) that has been related to diverse metabolic or aging-related diseases [23, 24]. The main UPR signaling cascades are initiated by three ER-localized protein sensors: inositol-requiring enzyme 1 (IRE1), protein kinase RNA-like endoplasmic reticulum kinase (PERK), and activating transcription factor 6α (ATF6α). ER stress-activated IRE1 stimulates c-Jun N-terminal kinase (JNK), which, in turn, phosphorylates serine residues in IRS-1, thereby inhibiting insulin receptor signaling [25] and limiting the activation of PI3K/Akt signaling in response to insulin. The reduced Akt activity leads to elevated nuclear FoxO activity. Understanding the mechanisms of FoxO1 action is of great importance as it may allow for the identification of new therapeutic targets for age-associated hepatic steatosis. Although detailed information is available on the molecular mechanisms underlying hyperglycemia and lipid accumulation, their relations with the presumed insulin signaling and FoxO1, remain unknown.

In the present study, we investigated hepatic steatosis in relation to hyperglycemia in the context of aging, as well as FoxO1 interaction with PPARγ during ER stress, in both the liver and AC2F cells, to achieve a better understanding of the molecular mechanisms involved in hepatic lipogenesis.

## RESULTS

### Changes in glucose and lipid metabolism in the aging liver

**Figure 1:**
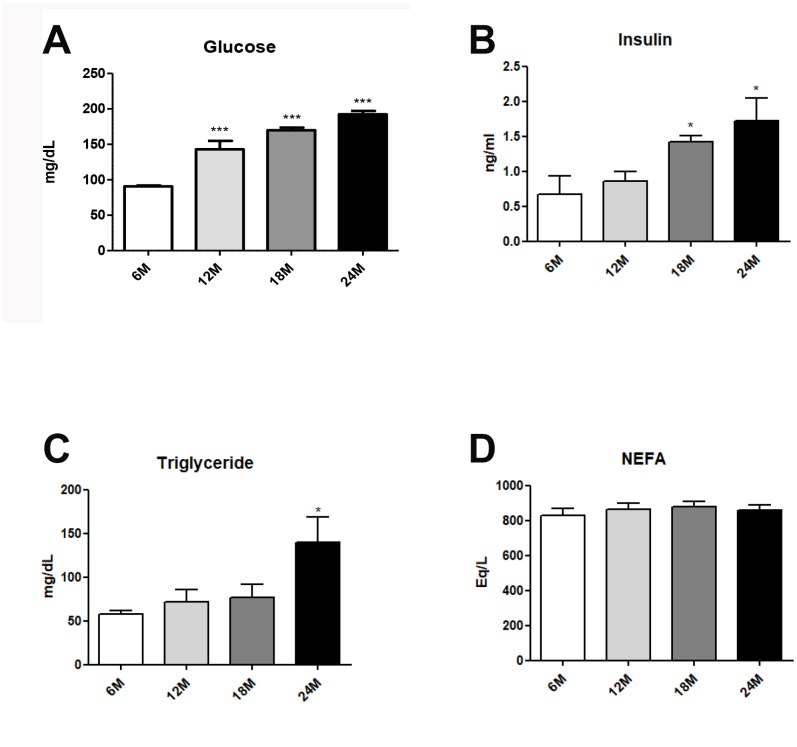
**Changes of aging-related serum parameter in insulin resistance and lipogenesis.** (**A**) Fasting glucose levels (**B**) insulin levels (**C**) triglycerides (**D**) FFA level in the serum of aging rats (each *n* = 6). Results of one- factor ANOVA: ^*^p < 0.05, ^***^p < 0.001 vs. 6-months old rats.

Obesity generally accompanies alterations in the blood lipid profile, which are closely associated with hepatic steatosis [26]. Blood lipid profiles were investigated in aged rats. Age-related hyperglycemia was significantly elevated fasting glucose (Figure 1A). However, hyperinsulimia and hypertriglyceridemia significantly elevated insulin levels (Figure 1B), and plasma TG levels (Figure 1C), as compared to age/sex-matched young animals. No age-related differences were found in the levels of plasma free fatty acid (NEFA) (Figure 1D). Our data strongly suggest that aging induces dyslipidemia and hyperglycemia.

### Age-related alterations in ER stress and insulin signaling

**Figure 2:**
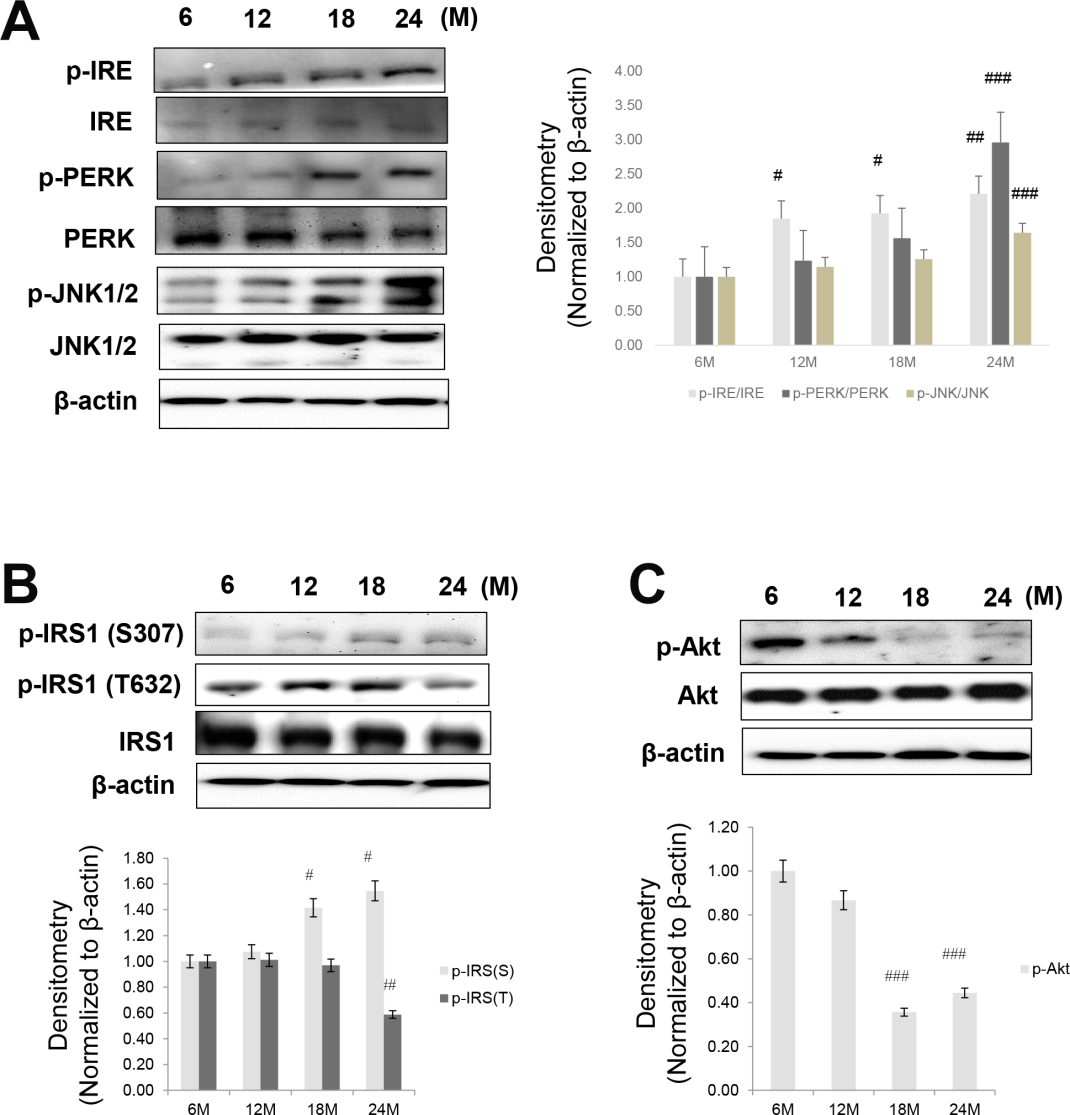
**Aging-related increase in ER stress and insulin signaling.** Western blotting was performed to detect the protein level of factors involved in ER stress, insulin signaling, and Akt signaling. (**A**) ER stress markers (p-IRE, IRE, p-PERK, PERK, p-JNK, and JNK) (**B**) insulin signaling factors (pSer-IRS1, pTyr-IRS1, IRS1) (**C**) aging-related increase in phospho-Akt level. β-actin was the loading control of the cytosolic fractions. Results of one-factor ANOVA: ^#^*p* < 0.05, ^##^*p* < 0.01, and ^###^*p* < 0.001 vs. 6 months.

ER stress significantly contributes to the development of insulin resistance by impairing insulin signaling through the activation of JNK, followed by phosphorylation of Ser307 in IRS1 [27]. In our study, the protein levels of ER stress markers including p-PERK, p-IRE, and p-JNK were dramatically increased in aged animals (Figure 2A).

Since fasting glucose levels are primarily regulated by insulin in the liver, we examined insulin signaling in the livers of aged rats. As shown in Figure 2, p-IRS1 (Ser307), a marker of insulin resistance, was increased in these animals, whereas the levels of Tyr632-phosphorylated IRS1 and Ser473-phosphorylated Akt were decreased with aging (Figure 2B and 2C). This suggests that ER stress increased and suppressed insulin signaling in the aging liver.

### Modulation of PPARs and FoxO1 activation in the aging liver

**Figure 3:**
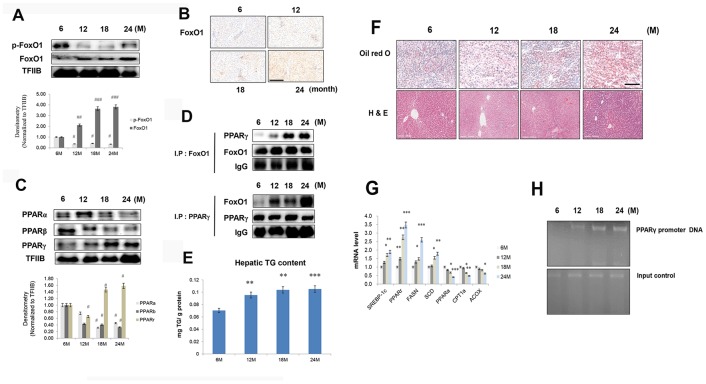
**Aging-related increase in FoxO1-induced lipid accumulation**. (**A**) Western blotting was performed to examine the protein levels of p-FoxO1 and FoxO1 in the liver of aging rats. Results of one-factor ANOVA: ^##^*p* < 0.01, and ^###^*p* < 0.001 vs. 6 months. (**B**) Immunohistochemical staining for FoxO1 in aging liver. Scale bar: 200 μm. (**C**) Western blotting analysis of PPARs in the nuclear of aging liver. TFIIB was the loading control of the nuclear fraction. One representative result of the three experiments for each protein is shown. Results of one-factor ANOVA: ^#^*p* < 0.05 vs. 6 months. (**D**) Western blotting showed that immunoprecipitated FoxO1 and PPARγ were physically associated with PPARγ and FoxO1, respectively. (**E**) Hepatic TGs in aging rats. Results of one-factor ANOVA ^**^p < 0.01, and ^***^p < 0.001 vs. 6 months. (**F**) Aging livers were stained with Oil red O to visualize lipid accumulation. Scale bar: 100 μm. Representative H&E staining shows increased vacuoles in liver tubules during aging. Scale bar: 300 μm. (**G**) Real-time PCR analyses was performed for measuring the mRNA levels of SREBP-1c, PPARγ, FASN, SCD, PPARα, CPT1α, and ACOX. The data are expressed as a mean ± SEM. ^*^p < 0.05, ^**^p < 0.01, and ^***^p<0.001 vs. 6 months. (**H**) FoxO1 binds to the PPARγ promoter in aging livers. The livers were subjected to ChIP assay by using rabbit pre-immune IgG and an anti-FoxO1 antibody. Immunoprecipitates were subjected to PCR by using rat PPARγ promoter DNA.

FoxO1 is a transcription factor that is mainly inhibited by Akt under insulin signaling. It contains three Akt-controlled phosphorylation sites, namely, Thr24, Ser256, and Ser319. FoxO1 dephosphorylation enhances its stability and activity, thereby stimulating gluconeogenesis and hyperglycemia. Phosphorylation of FoxO1 by Akt promotes FoxO1 translocation from the nucleus to the cytoplasm, preventing its activity as a transcription factor [28]. We found that in the livers of aged rats, FoxO1 phosphorylation was reduced (Figure 3A), whereas its expression was increased, as assessed by immunohistochemical staining (Figure 3B).

PPARs have been shown to modulate ER stress, insulin resistance, and inflammasome formation [29–32], all processes that are elevated with aging. To examine these phenomena in aged rats, western blotting was performed on liver homogenates. Nuclear protein levels of PPARα and PPARβ were reduced in the livers of aged rats compared to those of young rats. On the other hand, nuclear PPARγ level was increased in aged animals (Figure 3C), as also confirmed by immunohistochemical staining in liver (Supplementary Figure 1). In addition, our immunoprecipitation experiments showed that an interaction between FoxO1 and PPAR**γ** was induced in the aged liver (Figure 3D). To verify possible age-related changes in lipid accumulation, we measured the content of TGs in liver homogenates. The concentration of hepatic TGs was notably increased in aged animals (Figure 3E). These results were confirmed by liver Oil red O staining (Figure 3F). Moreover, we detected an overall age-related vacuolization in liver tubules (Figure 3F). Next, the role of FoxO1 as a regulator of lipogenesis genes was explored in relation to the aging process. Aged rats exhibited remarkably high mRNA levels of PPARγ as well as its target genes, such as FASN, SCD, and SREBP-1c (Figure 3G). However, the transcripts of β-oxidation genes such as PPARα, CPT1α, and ACOX were notably decreased in these animals (Figure 3G). In chromatin immunoprecipitation assay, FoxO1 associated with PPARγ promoter DNA in aged liver and exhibited a stimulatory effect on PPARγ expression compared with young rats (Figure 3H). These data indicated that, in the aging liver, the interaction between FoxO1 and PPARγ induces PPARγ transcriptional activity, upregulating lipogenesis genes.

### ER stress mediates lipid accumulation through FoxO1-induced PPARγ

**Figure 4:**
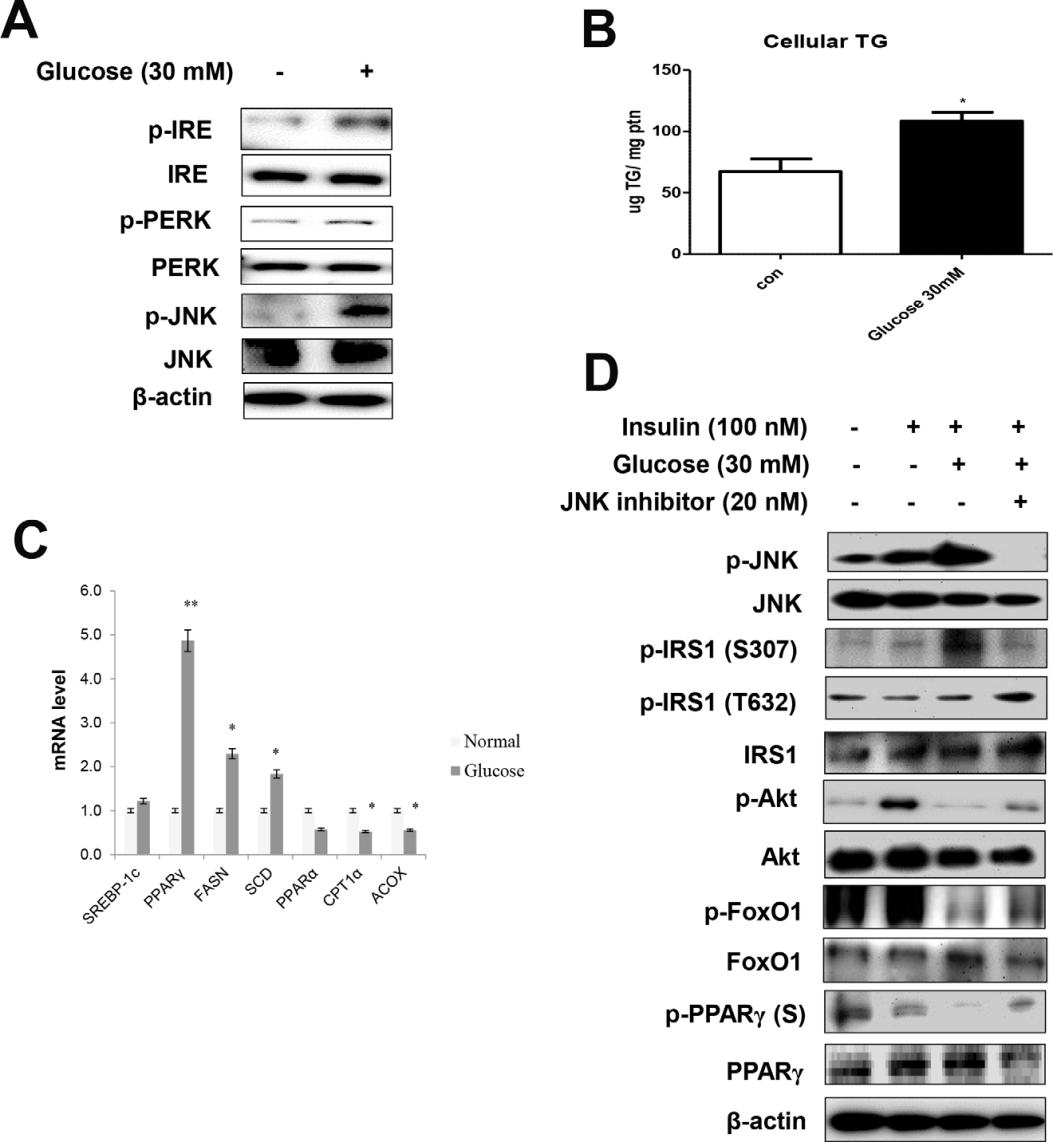
**High glucose induced ER stress-mediated lipid accumulation.** (**A**) Western blot was used to detect p-IRE, total-IRE, p-PERK, total-PERK, p-JNK, and total-JNK in cytoplasmic extracts (20 μg protein) after treatment of AC2F cells with glucose (30 mM) for 6 h. β-actin was the loading control of the cytosolic fractions. (**B**) Cellular triglyceride concentration after treatment with glucose (30 mM) for 36 h was measured by a colorimetric assay. The data are expressed as a mean ± SEM. Three independent experiments were performed and similar results were obtained. ^*^p < 0.05 vs. non-treated cells. (**C**) Real-time PCR analyses was performed for measuring the mRNA levels of lipogenesis genes (SREBP-1c, PPARγ, FASN, SCD) and β-oxidation genes (PPARα, CPT1α, and ACOX). The data are expressed as a mean ± SEM. Three independent experiments were performed and similar results were obtained. ^*^p < 0.05, and ^**^p < 0.01 vs. non-treated cells. (**D**) After stimulation with glucose (30 mM) for 2 h with insulin (100 nM) for 10 min in the absence (-) or presence (+) of JNK inhibitor (PD98059, 20 μM) for 1 h, cells were lysed and analyzed by western blotting. β-actin was the loading control of the cytosolic fractions.

We further explored the relationships between age and ER stress. To this end, serum-starved AC2F cells were treated with 30 mM glucose. The treatment enhanced the ER stress markers (Figure 4A) and induced cellular TGs (Figure 4B). These results were confirmed by the evaluation of mRNA levels. Glucose treatment significantly increased the transcription of lipogenesis genes in liver cells (Figure 4C). Furthermore, high-glucose treatment induced a decrease in cell viability, suggesting a role of hyperglycemia in the induction of lipotoxicity under these stress conditions (Supplementary Figure 2).

We examined the effects of ER stress activator and JNK inhibitor, PD98059, on insulin signaling, as well as FoxO1 and PPAR**γ** phosphorylation. In these experiments, serum-starved AC2F cells were treated with PD98059 for 1 h prior to a 2 h treatment with 30 mM glucose with insulin (100 nM) for 10 min. Glucose enhanced ER stress, whereas the JNK inhibitor prevented glucose-induced FoxO1 dephosphorylation and insulin signaling and increased the phosphorylation of both FoxO1 and PPARγ through JNK signaling (Figure 4D). We further verified whether the ER stress activator, tunicamycin, affected FoxO1 and PPARγ expression (Supplementary Figure 3) in AC2F cells and found that ER stress-mediated FoxO1 induced lipid accumulation. Furthermore, siRNA-mediated FoxO1 knockdown markedly decreased lipid accumulation in AC2F cells. Taken together, these findings provide evidence that ER stress increase the expression of FoxO1 and PPARγ, leading to lipid accumulation.

### FoxO1 activation increases ER stress in liver cells

**Figure 5:**
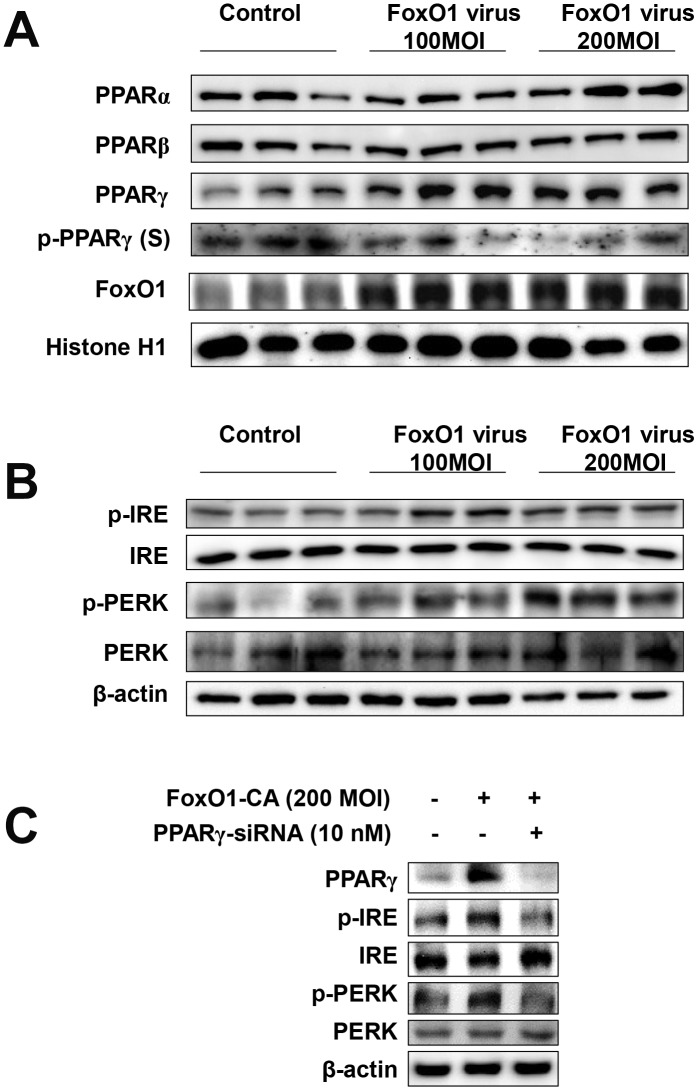
**FoxO1 regulates ER stress through PPARγ in FoxO1-virus treated cells.** (**A**) Activation of PPARγ by FoxO1. AC2F cells were grown to 80% confluence in 100 mm dishes in DMEM, and then stimulated with 100 and 200 MOI FoxO1 and analyzed by western blotting using the appropriate antibody. (**B**) FoxO1-induced activation of ER stress genes. Western blot was used to detect p-IRE, total-IRE, p-PERK, and total-PERK in cytoplasmic extracts (20 μg protein) from AC2F cells. (**C**) AC2F cells were grown to 80% confluence in 100-mm dishes containing DMEM, pretreated (one day) with or without PPARγ-siRNA (10 nM), then stimulated with the FoxO1 virus (200 MOI) for 1 days, and Western blot was used to detect PPARγ, p-IRE, total-IRE, p-PERK, and total-PERK in cytoplasmic extracts (20 μg protein) by using the β-actin as a control from AC2F cells.

To determine whether FoxO1 plays a role in the functional relationships between lipogenesis genes and aberrant ER stress, we employed a viral system for the exogenous overexpression of FoxO1 in AC2F cells. AC2F cells were intravenously transfected with Adv-FoxO1-CA or empty vector and ER stress was assessed. We examined the expression of ER stress genes in FoxO1 virus-transduced AC2F cells. Cells were treated with or without different concentrations (100 and 200 MOI) of FoxO1-CA. As shown in Figure 5A, treatment with 100 and 200 MOI FoxO1 induced PPAR**γ** levels. However, β-oxidation regulated transcriptional factors such as PPARα and PPARβ were not affected in FoxO1-transduced hepatocytes (Figure 5A). To verify the hypothesis that FoxO1 transactivates the ER UPR proteins, the expression of these genes was assessed in FoxO1 virus-transduced AC2F cells. ER UPR proteins were found to be induced in the FoxO1-overexpressing but not the mock-transfected cells. Taken together, these results indicated that FoxO1 promotes the activation of the ER UPR proteins, PERK and IRE (Figure 5B). We examined the expressions of ER stress genes under condition of FoxO1 overexpression with PPAR**γ-**siRNA knockdown in cells. As shown in Figure 5C, overexpression of FoxO1 induced ER stress gene levels. Otherwise, ER stress genes were reduced by FoxO1 overexpression with PPAR**γ**-siRNA. Collectively, these data indicate that PPAR**γ** induced ER stress genes suggesting that FoxO1-mediated PPARγ may lead to ER stress.

### Interaction between FoxO1 and PPARγ in liver cells

**Figure 6:**
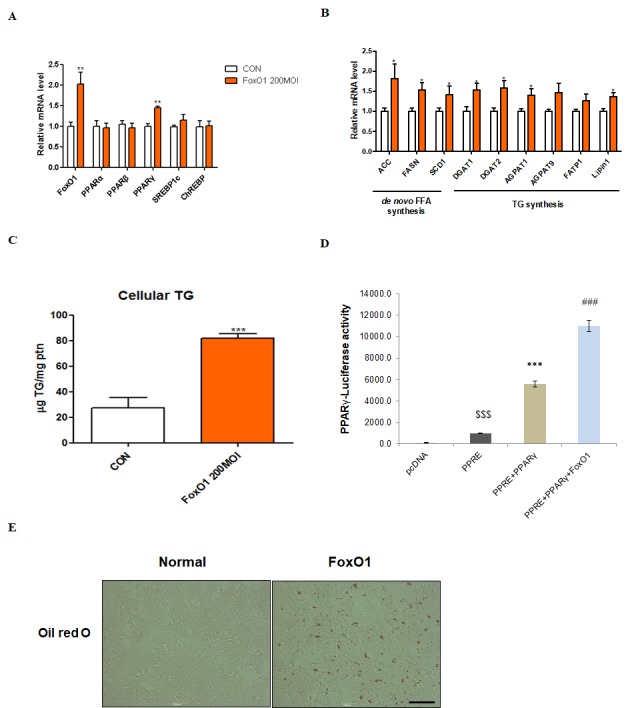
**FoxO1-dependent changes in lipid accumulation in liver cells.** (**A**) AC2F cells incubated with or without FoxO1 (200 MOI) for 24 h were subjected to real-time qRT–PCR analysis of different transcripts (FoxO1, PPARα, PPARβ, PPARγ, SREBP-1c, and ChREBP) by using the β-actin gene as a control. Results of one-way ANOVA: ^**^p < 0.01 vs. untreated cells. (**B**) Cells incubated without or with FoxO1 (200 MOI) for 24 h were subjected to real-time qRT-PCR analysis of different transcripts (ACC, FASH, SCD1, DGAT1, DGAT2, AGPAT1, AGPAT9, FATP1, and lipin1) by using the β-actin gene as a control. Results of one-way ANOVA: ^*^p < 0.05 vs. untreated cells. (**C**) Cellular triglyceride concentration was measured by a colorimetric assay. The data are expressed as a mean ± SEM. Three independent experiments were performed, providing similar results. ^***^p < 0.001 vs. untreated cells. (**D**) Effect of wild-type FoxO1 on the activity of the PPARγ promoter. AC2F cells in 48-well microplates were transduced with AdV-FoxO1 or control AdV-null vectors at a fixed dose (MOI, 200 pfu/cell), followed by transfection with 1 μg of pcDNA and PPARγ DNA in the culture medium. After a 24 h incubation, the cells were harvested. The relative luciferase activity was calculated based on the PPARγ-luciferase/β-galactosidase activity ratio. The data are expressed as a mean ± SEM. Three independent experiments were performed and similar results were obtained. ^$$$^p < 0.001 vs. pcDNA treated cells; ^***^p < 0.001 vs. PPRE treated cells; ^###^p < 0.001 vs. PPRE with PPARγ DNA treated cells. (**E**) FoxO1 transfected cells were stained with Oil red O to visualize lipid accumulation. Scale bar: 100 μm.

We used the AC2F liver cell line to further investigate the functional role of FoxO1 in hepatic steatosis. We hypothesized that FoxO1-induced lipid accumulation in the liver occurs, at least in part, via PPARγ activation. As a transcription factor, PPAR**γ** plays an important role in hepatic steatosis by inducing lipogenesis-related gene expression [33–35]. We verified whether FoxO1 induces PPARγ mRNA in AC2F cells. Notably, no change in the expression of other transcription factors related to lipid metabolism, such as PPARα, PPARβ, SREBP-1c, and ChREBP, was detected (Figure 6A). However, FoxO1 adenoviral overexpression markedly increased the transcription of target genes such as ACC, FASH, SCD1, DGAT1, DGAT2, AGPAT1, AGPAT9, FATP1, and lipin1 (Figure 6B). We explored the effect of FoxO1 overexpression on lipid accumulation in AC2F cells and found a marked FoxO1-dependent increase in TG concentration (Figure 6C). Moreover, FoxO1 caused the transcriptional activation of PPARγ, as determined by PPARγ luciferase assay (Figure 6D). These results were confirmed by liver cell Oil red O staining, showing that fat accumulates in FoxO1-overexpressing cells (Figure 6E). Based on these findings, we suggest that FoxO1 induces lipid accumulation through the upregulation of lipogenesis genes.

### Modulation of hepatic TGs in FoxO1-siRNA transfected cells

To further establish the importance of FoxO1 in ER stress, we employed siRNA-mediated gene-silencing to knock down FoxO1 expression in AC2F cells.

**Figure 7:**
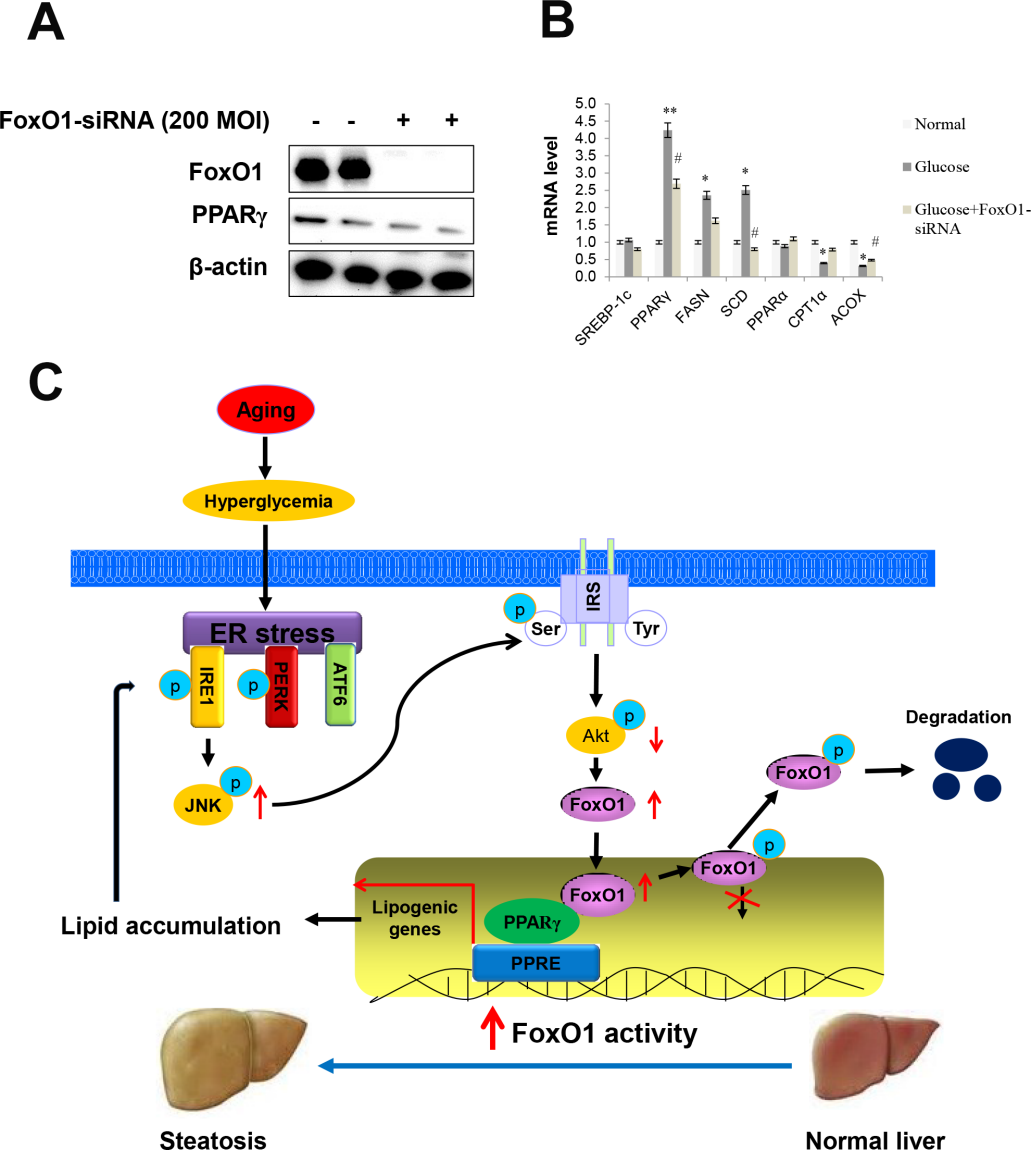
**Effect of high glucose and FoxO1 deletion on the regulation of lipid metabolism.** (**A**) Western blot was used to detect FoxO1 and PPARγ in FoxO1-siRNA treated liver cells. β-actin was used as a loading control. (**B**) The expression of SREBP-1c, PPARγ, FASN, SCD, PPARα, CPT1, and ACOX was analyzed by qPCR after treatment with glucose (30 mM) for 24 h in FoxO1-siRNA transfected (200 MOI) cells. The results were normalized based on the actin level. (**C**) Possible mechanism by which FoxO1 activates ER stress-induced lipogenesis in aging.

First, we examined the effect of the phosphoinositide 3-kinase (PI3k)/Akt pathway on FoxO1 phosphorylation, by utilizing a constitutively active Akt (CA-Akt), and evaluated Akt-dependent phosphorylation of FoxO1 and PPARγ (Supplementary Figure 4). We also measured FoxO1 and PPARγ levels by western blotting. Significant differences in the expression levels of FoxO1 and PPARγ were observed in FoxO1-deficient cells (Figure 7A). However, high glucose notably increased the transcription of various genes including PPARγ, FASN, and SCD, which, in turn, led to decreased expression of lipogenesis genes, as determined by q-PCR analysis in the glucose-treated FoxO1-deficient cells (Figure 7B). Liver cell Oil red O staining confirmed decreased fat accumulation in FoxO1 deficient cells (Supplementary Figure 5). Taken together, these results suggested that knockdown of FoxO1 partially prevented hyperglycemia-induced activation of lipogenesis genes.

### Relationship between ER stress and hepatic steatosis with insulin resistance

**Figure 8:**
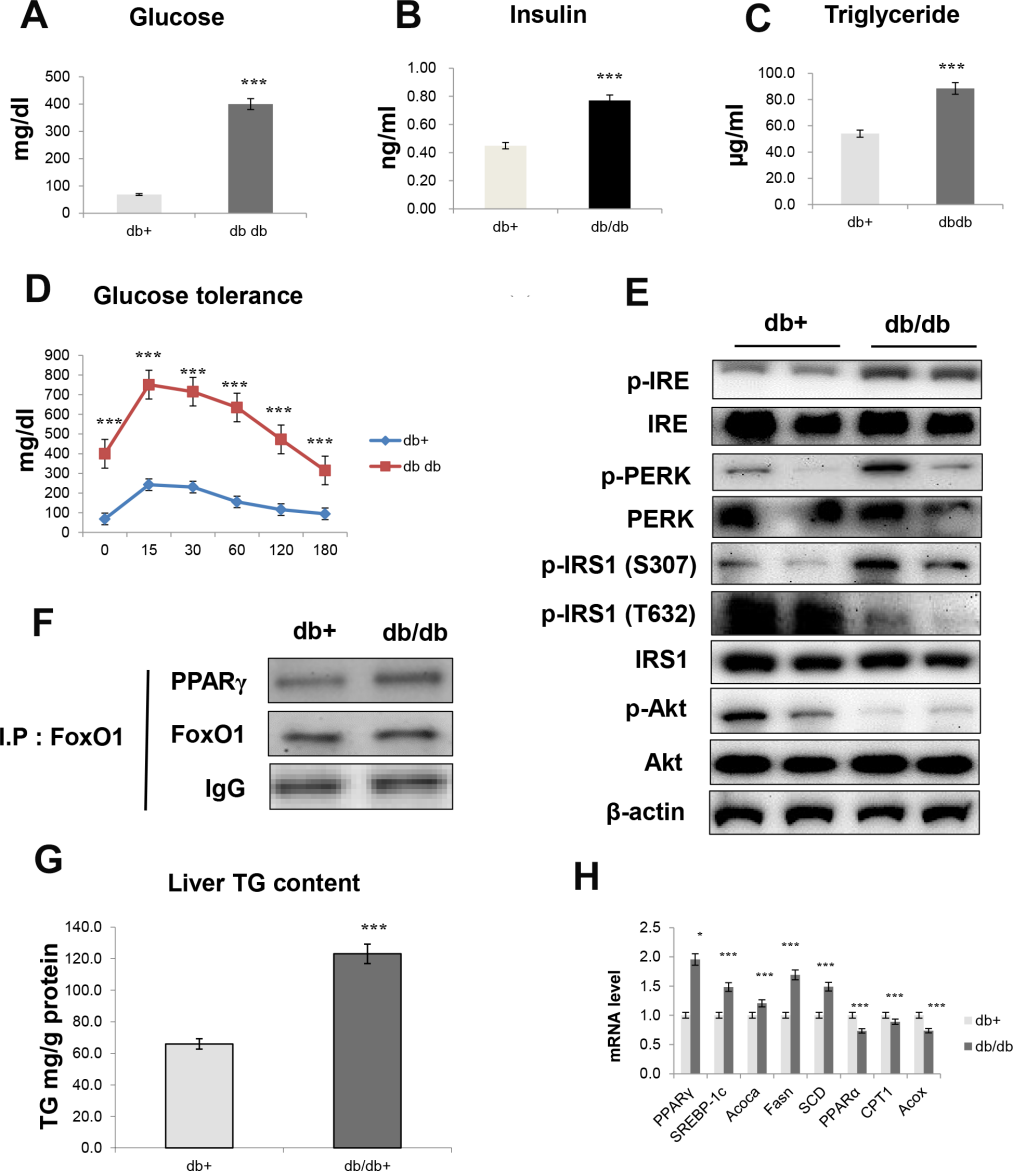
**Obesity induces hepatic steatosis and insulin resistance through ER signaling.** (**A**) Glucose level (**B**) insulin level (**C**) triglyceride level (**D**) glucose tolerance test in the serum of obese models. The data are expressed as a mean ± SEM (each n=5). The data are expressed as a mean ± SEM. ^***^p<0.001 vs. db+. Western blot analyses of liver cytosolic (**E**) p-IRE, IRE, p-PERK, PERK, p-IRS1 (S307), p-IRS1 (T632), IRS1, p-Akt, and total-Akt levels were performed using cytosolic proteins from obese mice (*n* = 5 in each group). β-actin was the loading control of the cytosolic fractions. (**F**) Western blotting showed that immunoprecipitated FoxO1 and PPARγ were physically associated with PPARγ and FoxO1, respectively. (**G**) Hepatic triglyceride concentration was measured by a colorimetric assay. The data are expressed as a mean ± SEM. ^***^p<0.001 vs. db+. (**H**) PPARγ, SREBP-1c, Acoca, Fasn, SCD, PPARα, CPT1, and Acox mRNA. Real-time PCR analyses were performed to determine the mRNA levels in liver tissues of db/db mice (*n* = 5 in each group). The data are expressed as a mean ± SEM. ^*^p < 0.05, and ^***^p<0.001 vs. db+.

In aged animals, insulin resistance was significantly increased and correlated with the pathogenesis of fasting hyperglycemia. We studied insulin resistance and hepatic steatosis in db/db mice. As shown in Figure 8, obese mice developed hyperinsulinemia, culminating in substantially higher fasting glucose (Figure 8A), insulin levels (Figure 8B), TG levels (Figure 8C), and glucose tolerance (Figure 8D), as compared to age-matched controls. Notably, insulin resistance models exhibited significantly increased expression of IRE and PERK, which are all key genes in ER stress. Furthermore, IRS/Akt signaling was suppressed in the liver of db/db mice (Figure 8E). In addition, our IP experiments showed that an interaction between FoxO1 and PPAR**γ** was triggered in the liver of obese mice (Figure 8F).

We examined whether obesity affected lipid accumulation in vivo and found that insulin resistance markedly increased TG concentration (Figure 8G). Moreover, we found that obesity induced lipogenesis genes in db/db mice, as demonstrated by the increased transcription of genes such as PPARγ, SREBP-1c, ACOCA, FASN, and SCD (Figure 8H). On the other hand, obesity decreased the transcription of β-oxidation genes, such as PPARα, CPT1, and ACOX (Figure 8H).

## DISCUSSION

Our study focused on the altered interaction between FoxO1 and PPARγ that independently mediate hepatic lipogenesis during age-related ER stress. It has been reported that FoxO1 improves hepatic insulin signaling and fatty liver disease, although the underlying mechanisms remain to be elucidated [36, 37]. Here, we examined the molecular mechanism by which FoxO1 ameliorates hepatic steatosis in aging. The lipotoxic environment of non-alcoholic fatty liver disease (NAFLD) is due to a surplus of lipids that directly influences ER homeostasis and ER stress activation [38]. To date, little is known about FoxO1 regulation of ER stress-induced lipogenesis. Our data suggested that aging induces insulin signaling via ER stress (Figure 2). We found that, during insulin signaling, the level of Akt protein decreased and FoxO1 was dephosphorylated. ER stress and insulin resistance have not been well investigated in aging. Aging was associated to impaired Akt/FoxO1 signaling and ER stress normalization. We assumed that the changes in glucose and lipid metabolism are partially mediated by ER stress in the aging liver. The ER is particularly susceptible to protein misfolding. Proteins that are unable to fold correctly, because of alterations in the physiological and molecular environment, cause ER stress and activate the UPR [39]. ER stress is closely related to metabolic syndrome in aging [40], partially as a result of JNK pathway activation, followed by IRS phosphorylation at Ser307 and the subsequent development of insulin resistance [41, 42]. Therefore, chemical or natural compounds that alleviate ER stress may act as potential insulin-sensitizing agents against aging-induced metabolic syndrome.

FoxO transcription factors are pivotal downstream targets of insulin/IGF-1 signaling and have been postulated to influence longevity by conferring increased resistance to oxidative stress, decreasing ROS production, and slowing the accumulation of oxidative damage, factors known to modulate aging process [43, 44]. Some of these anti-oxidative effects are mediated by members of the FoxO family, which in the absence of insulin/IGF-1 signaling bind to promoters of antioxidant enzymes and upregulate their expression [45]. These and other findings strongly suggest the involvement of FoxO in the aging process and age-related diseases. For example, FoxO reduces the toxicity associated with aggregation-prone mutant proteins involved in human Alzheimer’s and Huntington’s disease, which suggests that the regulation of homeostasis during aging has a direct effect on the pathogeneses of human neurodegenerative diseases [46–48].

The protein level of the lipogenesis transcription factor PPARγ was induced by the aging process (Figure 3). It is known that FoxO1 and PPARγ are closely associated with hepatic steatosis [33, 36, 49] and our study provides additional evidence that ER stress induces hepatic steatosis through FoxO1-induced PPARγ upregulation. The overexpression of FoxO1 was shown to stimulate hepatic PPARγ expression in cells (Figure 5). Our data indicated that adenoviral overexpression of FoxO1 increased the transcription of lipogenesis genes such as ACC, FAS, and SREBP-1c in AC2F cells (Figure 6). Consistently, TG accumulation was increased under these conditions, thus supporting a role for FoxO1 in enhanced hepatic lipid accumulation through increased lipogenesis. It is well documented that PPARγ stimulates hepatic lipid accumulation [33–35, 50]. In genetically and diet-induced obese mice, increased PPARγ expression is involved in hepatic steatosis by increasing the expression of lipogenesis gene [33, 34, 50]. In line with these findings, liver-specific deletion of PPARγ in db/db mice markedly ameliorated hepatic steatosis by downregulating FAS, SCD1, and ACC [35]. Although various studies reported [51] that both FoxO1 and PPARγ stimulated hepatic steatosis, the functional relationship between these two proteins in the context of aging is unclear. Our data showed that FoxO1 increased PPARγ transcriptional activity, as assessed by peroxisome proliferator-responsive element (PPRE) luciferase assay (Figure 6D). These results also indicated that the FoxO1-mediated increase in PPARγ activity contributes to hepatic lipid accumulation. Further studies are necessary to establish whether FoxO1 also controls PPARγ activity in other metabolic organs, such as adipose tissue and skeletal muscle, to regulate lipid metabolism. In addition, free fatty acids significantly activate ER stress-related gene expression in AC2F cells. FoxO1 expression and activity have been reported to be increased in human steatohepatitis livers and are correlated with the severity of nonalcoholic steatohepatitis [52]. Moreover, ER stress is activated under conditions of cellular nutrient overload, including lipotoxicity [53].

We have shown that the expression of lipogenesis genes was increased in aging-related db/db mice and that this change was due to increased expression of the transcription factors FoxO1 and PPARγ, both involved in lipid accumulation (Figure 8). We confirmed that ER stress-mediated hepatic steatosis is modulated by FoxO1 through insulin signaling in aged db/db mice. However, little is known on the protective mechanisms induced by FoxO signaling and their possible dependence on tissue type, age, or disease context. The ability of FoxO to regulate inflammation plays a central role in relation to many metabolic disorders and diseases, such as obesity, type 2 diabetes mellitus, insulin resistance, hyperlipidemia, and NAFLD [54]. Although its incidence is age-related, metabolic syndrome is associated with a high-fat diet and excessive calorie intake, independently of age [55]. The clear association of metabolic syndrome with aging indicates that inflammation is crucial process for disease onset and progression [56].

In summary, the reciprocal activation ER stress attenuated Akt signaling by a major mechanism underlying glucose-mediated hepatic lipogenesis and is caused by an altered interaction between FoxO1 and PPARγ during aging (Figure 7C). Our results reveal significant insights into the cellular and molecular basis of the FoxO1/PPARγ association, proposing this complex as a novel candidate target for the treatment of altered lipogenesis.

In conclusion, hyperglycemia-induced ER stress causes Akt inhibition, which activates FoxO1. FoxO1 interaction with PPARγ leads to hepatic lipogenesis *in vivo* and *in vitro*. In our future studies, we will focus on the potential application of these findings in the prevention of liver diseases and associated complications.

## MATERIALS AND METHODS

### Materials

Western blotting detection reagents were obtained from Amersham (Bucks, UK). Antibodies against FoxO1, β-actin, istone H1, TFIIB, p-Akt, and Akt were obtained from Santa Cruz Biotechnology (Santa Cruz, CA, USA). An antibody against p-FoxO1 (Ser-256) was obtained from Cell Signaling Technology, Inc. (Danvers, MA, USA). An anti-rabbit IgG–horseradish peroxidase (HRP)-conjugated antibody and an anti-mouse IgG–HRP-conjugated antibody were obtained from Amersham Pharmacia Biotech (Bucks, UK). An HRP-conjugated donkey anti-sheep/goat IgG was purchased from Serotec (Oxford, UK). Polyvinylidene difluoride (PVDF) membranes were obtained from Millipore Corporation (Bedford, MA, USA). JNK inhibitor (PD98059) was obtained from Sigma-aldrich (St. Louis, Missouri, USA). Virus against FoxO1, FoxO1-siRNA and CA-Akt were obtained from Dr. HH Dong (University of Pittsburgh, Pittsburgh, PA, USA.

### Animals

Male Sprague-Dawley (SD) rats (aged 6, 12, 18, and 24 months) were obtained from Samtako (Osan, South Korea). SD rats groups (n=6/group) were given water and a standard laboratory diet *ad libitum* (AL). Eight-week-old male C57BLKS/J-lean and C57BLKS/J-db/db (diabetic) mice were purchased from Japan SLC, Inc (Hamamatsu, Japan). The mice were maintained under a 12 h light/dark cycle at 23 ± 1 °C and 50 ± 5% relative humidity under specific pathogen-free conditions. The animal protocol used in this study was reviewed and approved by the Pusan National University-Institutional Animal Care and Use Committee (PNU-IACUC) in compliance with the relevant ethical and scientific care procedures (PNU-2015-0944).

### Tissue homogenate

All solutions, tubes, and centrifuges were maintained at 4 °C. One gram of liver was homogenized with 700 μl of hypotonic lysis buffer [buffer A: 100 mM Tris (pH 7.4), 20 mM β-glycerophosphate, 20 mM NaF, 2 mM sodium orthovanadate, 1 mM EDTA, 0.01 mM DTT, 0.5 mM PMSF, 1 μM pepstatin, 2 μM leupeptin, and 10 mM HEPES (pH 7.8)] using a tissue homogenizer for 30 sec. After homogenates were kept on ice for 20 min, 125 μl of 10% Nonidet P-40 (NP-40) solution was added, mixed for 15 sec, and then centrifuged at 12,000 *g* at 4 °C for 15 min. The supernatants were used as the cytosol fraction. The pellets were washed with 300 μl of hypotonic buffer A plus 25 μl of 10% NP-40, centrifuged, suspended in 200 μl of buffer C [50 mM KCl, 300 mM NaCl, 1 mM dithiothreitol (DTT), 0.1 mM EDTA, 0.1 mM PMSF, 10% (v/v) glycerol, 1 μM pepstatin, 2 μM leupeptin, 20 mM β-glycerophosphate, 20 mM NaF, 2 mM Na-ortovanodate, and 50 mM HEPES (pH 7.8)], kept on ice for 30 min, and centrifuged at 14,000 *g* for 10 min. The supernatant containing nuclear proteins was collected and stored in aliquots at -80 °C until use. The protein concentration was measured by the bicinchoninic acid (BCA) assay method using bovine serum albumin (BSA) as a standard.

### Cell culture system

AC2F cells (rat hepatocellular carcinoma) were obtained from the ATCC (American Type Culture Collection, Rockville, MD, USA). AC2F cells were cultured in Dulbecco’s Modified Eagle Media (DMEM) (Nissui Co., Tokyo, Japan) supplemented with 5% heat-inactivated (56 °C for 30 min) fetal bovine serum (Gibco, Grand Island, NY, USA), 233.6 mg/mL glutamine, 72 μg/mL penicillin streptomycin, and 0.25 μg/mL amphotericin B, and adjusted to pH 7.4-7.6 with NaHCO_3_ in a 5% CO_2_ atmosphere. Cells were maintained at 37 °C in a humidified atmosphere containing 5% CO_2_.

### Western blot

Western blotting was carried out as described previously and lysed samples were boiled for 5 min with gel-loading buffer [0.125 M Tris-HCl, pH 6.8, 4% SDS, 10% 2-mercaptoethanol, and 0.2% bromophenol blue] at a ratio of 1:1. Equal amounts of protein were separated by sodium dodecyl sulfate-polyacrylamide gel electrophoresis (SDS-PAGE) using 6–17% acrylamide gels. The gels were subsequently transferred onto Immobilon-P transfer membrane (Millipore Corp, Bedford, MA, USA). The membranes were immediately placed in blocking buffer [5% non-fat dry milk in TBS-Tween (TBS-T) containing 10 mM Tris, 100 mM NaCl, and 0.1% Tween 20, pH 7.5] at room temperature (25 °C) for 1 h.

The membrane was washed in TBS-T buffer for 30 min and incubated with the appropriate primary antibodies at 4 °C overnight. After 30 min washing in TBS-T buffer, the membrane was incubated with a secondary antibody at room temperature for 1 h. Then, after washing in TBS-T buffer for 40 min, antibody labeling was detected using ECL as per manufacturer’s instructions and a radiographic film was exposed to the membrane.

### Hepatic lipid content

Liver tissue and cell samples (20 mg) were homogenized in 400-μl HPLC-grade acetone. After overnight incubation with agitation at room temperature, 50 μl aliquots of acetone-extracted lipid suspension were used to determine triglyceride concentrations, employing the Infinity triglyceride reagent (Thermo Electron). Hepatic lipid content was defined as mg of triglyceride per g of total liver or cellular proteins, as described earlier.

### RNA isolation and real time RT-PCR

RNA isolation from liver (20 mg) or AC2F cells (~2 × 10^6^ cells) was performed using the RNeasy Mini Kit (Qiagen, Hilden, Germany). Real-time polymerase chain reaction (RT-PCR) was performed to measure relative mRNA concentrations using the Roche LightCycler-RNA amplification kit (Roche Diagnostics, Indianapolis, IN, USA). All primers were purchased from Integrated DNA Technologies (Coralville, IA, USA).

### Immunoprecipitation (IP) assay

Tissue extracts were immunoprecipitated in a buffer containing 40 mM Tris-HCl (pH 7.6), 120 mM NaCl, 20 mM β-glycerophosphate, 20 mM NaF, 2 mM sodium orthovanadate, 5 mM EDTA, 1 mM PMSF, 0.1% NP40, leupeptin (2 μg/ml), aprotinin (1 μg/ml), and pepstatin A (1 μg/ml) [57]. Aliquots of cell extracts were centrifuged at 12,000 *g* at 4 °C for 15 min, incubated overnight at 4 °C with the correspondent antibody, and then incubated overnight at 4 °C with a 50% protein A-agarose slurry. After washing the immunoprecipitates three times with immunoprecipitation buffer, the immunoprecipitated proteins were analyzed by SDS-PAGE and western blotting, as described above.

### Histological analysis

Livers tissue and cells were fixed in 10% neutral formalin and paraffin-embedded sections were stained with hematoxylin and eosin (H&E staining). The Oil Red O (Sigma, O1391) staining was performed by previously described methods. Frozen samples were cut at the temperature and stained.

### Biochemical analysis of serum

Serum glucose was analyzed using kits from Bioassay Systems (Hayward, CA, USA). Specific kits were used, following the manufacturer’s instructions, to determine the concentrations of insulin (Shibayagi, Japan), NEFA, and TG (Shinyang, South Korea).

### Glucose tolerance test

Mice were fasted for 24 h, followed by intraperitoneal injection of glucose (2 g/kg). Blood glucose levels were measured before and after glucose injection by using a Glucometer Elite meter (Bayer, IN, USA).

### Transfection and luciferase assay

PPARγ activities were estimated using the PPARγ-Luc vector. Transfection was carried out using Lipofectamine 2000 (Invitrogen). Briefly, 1 × 10^4^ cells per well were seeded in 48-well plates. When cultured cells reached about 40 % confluence, they were treated with 1 μg DNA/0.5 μl Lipofectamine 2000 complexes in 500 μl normal media (5% serum contained) for 24 h and then treated with the FoxO1-CA (200 MOI). After incubation for 24 h, the cells were allowed to incubate for an additional 24 h. Luciferase activity in the cells was analyzed by the Steady-Glo Luciferase Assay System (Promega, Madison, WI, USA), and was measured using a luminometer (GENious, TECAN, Salzburg, Austria).

### Cytotoxicity assays

Cytotoxicity was determined by 3-(4,5-dimethylthiazol-2-yl)-2,5-diphenyltetrazolium bromide (MTT) from Aldrich Chemical Co. (Madison, WI). Cells were seeded into 96-well plates, incubated overnight for adherence, and subsequently exposed to 30 mM glucose for various periods of time. At the end of the treatment, MTT reagent dissolved in PBS was added to the medium (final concentration of 0.5 mg/ml) and the plates were incubated in the dark for 1 h. After incubation, the supernatant was removed and formazan crystals were dissolved in 100 μl of dimethyl sulfoxide (DMSO) with gentle agitation. The absorbance in each well was measured at 450 nm by using a spectrophotometer.

### Immunohistochemistry analysis

For immunostaining, liver sections were treated with 0.6% H_2_O_2_ in Tris-buffered saline (TBS; pH 7.5) to block endogenous peroxidase for 15 min at room temperature. Next, sections were incubated with TBS containing 0.1 % Triton X-100 and 3 % goat serum (TBS-TS) at 37 °C for 1 h and then with primary anti-FoxO1 or anti-PPARγ antibody (Santa Cruz, Texas, USA) in TBS-TS overnight at 4 °C. Sections were further incubated with secondary goat anti-Rabbit IgG-HRP antibody (Santa Cruz) at RT for 3 h, stained with diaminobenzidine (DAB) solution, mounted with Dako mounting medium (Dako, Glostrup, Denmark), and coverslips were put on the sample. Images were acquired using an Olympus IX71 microscope (Olympus, Tokyo, Japan).

### Chromatin immunoprecipitation (ChIP) assay

ChIP was used to study the interaction between FoxO1 and PPARγ promoter DNA in the cell. Cells were subjected to the ChIP assay using the anti-FoxO1 antibody and a ChIP assay kit (Upstate Biotechnology). The immunoprecipitates were analyzed using immunoblot analysis using rabbit anti-FoxO1, as well as a PCR assay to detect co-immunoprecipitated DNA using PPARγ promoter-specific primers (forward: 5′-CATGCAAGAAATGGTGCTAA-3′ and reverse: 5′-TATGAAGGAGAACACCTTCA-3′), which flank the consensus FoxO1-binding sites in the rat PPARγ promoters, respectively.

### Small interfering RNA-mediated gene silencing

To knockdown PPARγ in AC2F cells, we utilized scrambled or PPARγ small interfering (si)RNAs obtained from a commercial source (IDT). Transfection was carried out using the Lipofectamine 2000 reagent (Invitrogen, Grand Island, NY, USA). The cells were treated with scrambled or PPARγ siRNA–Lipofectamine complexes (10 nM) in Opti-MEM (Invitrogen) serum-free medium. After incubation for 4 h, the transfection medium was replaced with a fresh medium, and the cells were incubated for another 48 h, during which time they were treated at the indicated times with an adenoviral vector containing the FoxO1-encoding sequence.

### Statistical analysis

To analyze differences among three or more groups, one-way analysis of variance (ANOVA) was used. Differences in the means of individual groups were assessed by Bonferroni’s *post hoc* test. Student’s *t* test was used to analyze differences between two groups. Results are expressed as means ± S.E.M. P values < 0.05 were considered statistically significant. The analyses were performed using Graph Pad Prism 5 (Graph Pad software, La Jolla, CA, USA).

## Supplementary Material

Supplementary Figures
