# Supplementary material for: Altered FoxO1 and PPARγ interaction in age-related ER stress-induced hepatic steatosis
Source: Aging (Albany NY). 2019 Jun 25;11(12):4125–44. doi: 10.18632/aging.102042 (PMC6628996; doi:10.18632/aging.102042)
Supplement: Supplementary Figures [file aging-11-102042-s001.pdf]

SUPPLEMENTARY FIGURES

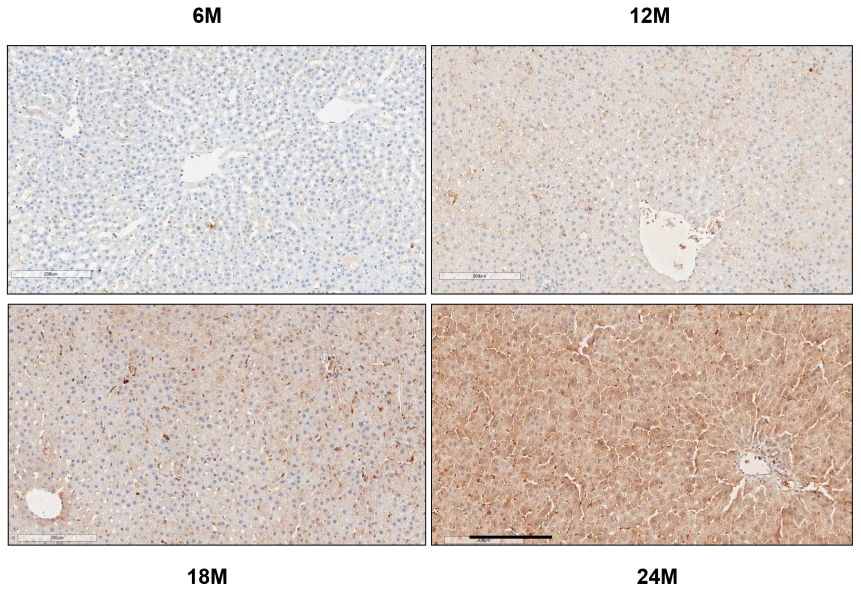

Supplementary Figure 1. Aging-related PPARγ changes. Immunohistochemical staining for PPARγ in aged liver. Scale bar: 200 μm.

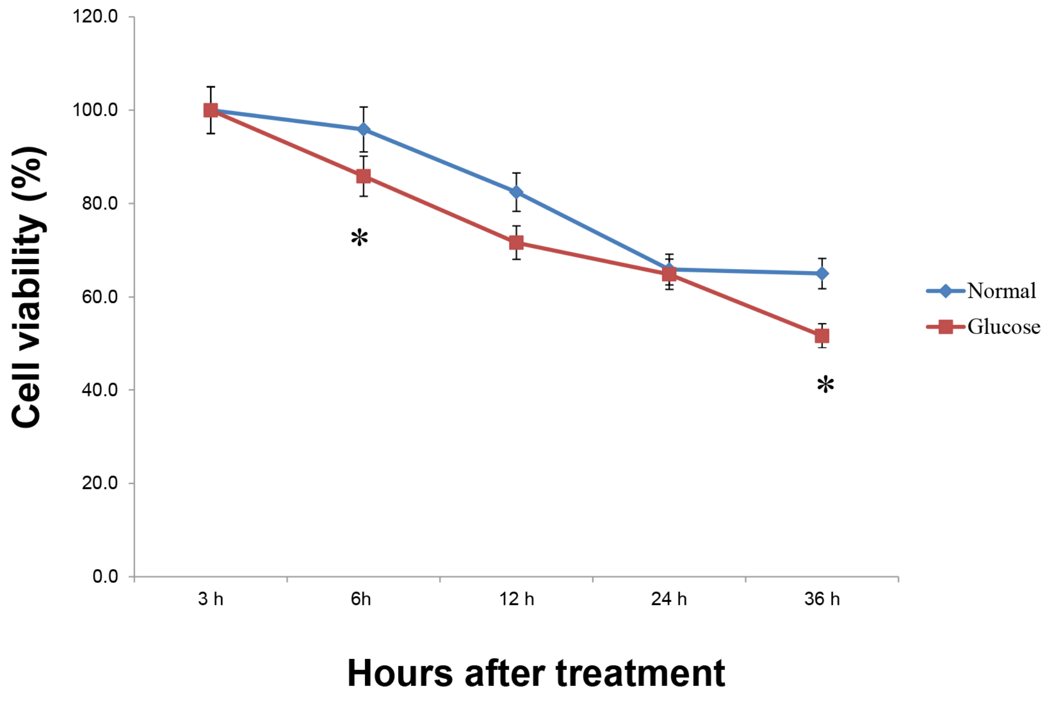

Supplementary Figure 2. Toxicity of high glucose in AC2F cells. After a 36-h treatment with glucose (30 mM), the cell viability was determined by MTT assay, as described in the Materials and Methods. Each bar is the mean ± SEM of three measurements. Statistical significance: *$p < 0.05$ vs. untreated group.

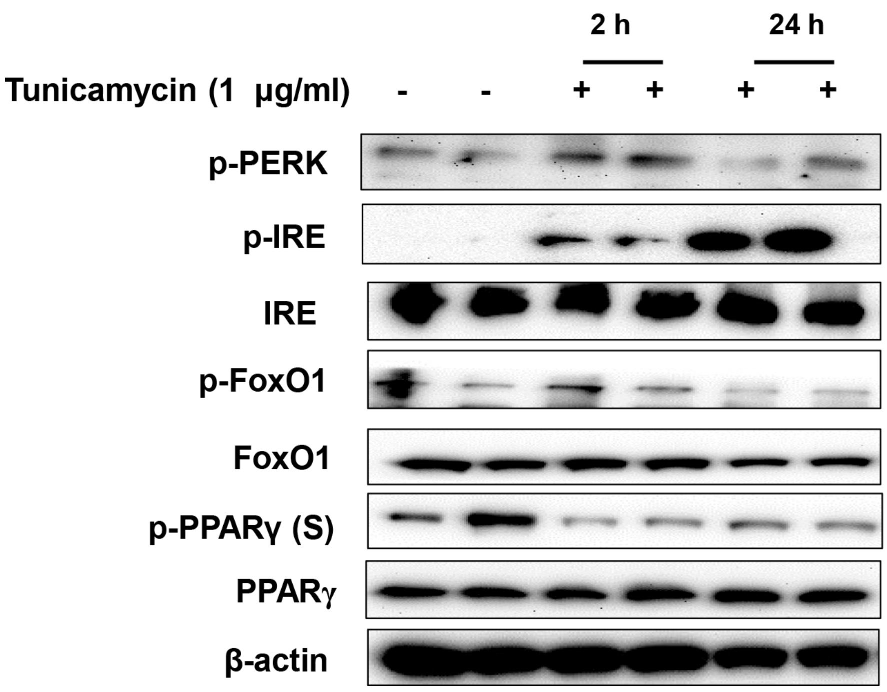

Supplementary Figure 3. ER stress activator induces lipid metabolism. Western blotting analysis of ER stress genes, FoxO1, and PPARγ in ER stress activator (Tunicamycin) for 2 h and 24 h in cells. Proteins were subjected to semiquantitative immunoblot analysis for for p-PERK, p-IRE, IRE, p-FoxO1, FoxO1, p-PPARγ, PPARγ, and β-actin was used as a loading control.

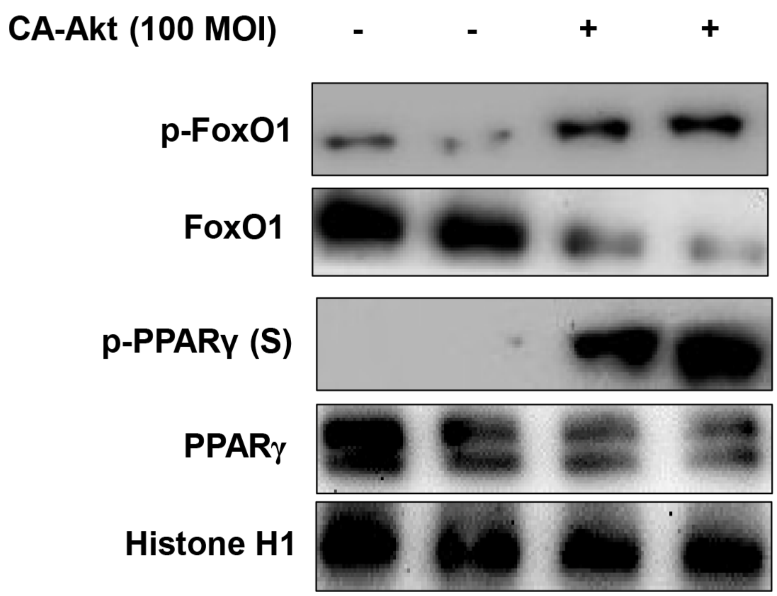

Supplementary Figure 4. Akt inhibits PPARγ activation in cells. AC2F cells were grown to 80% confluence in 100 mm dishes in DMEM, and then stimulated with 100 MOI Akt-CA for 24 h and analyzed by western blotting using the appropriate antibody**.** Histone H1 was the loading control of the nuclear fraction.

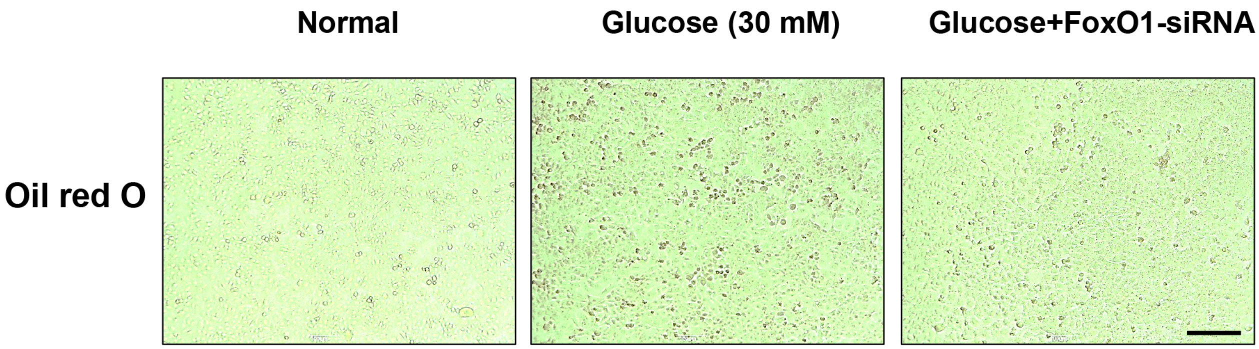

Supplementary Figure 5. Glucose induces lipid production through FoxO1 activation. Lipid accumulation was analyzed by Oil red O after treatment with glucose (30 mM) for 48 h in FoxO1-siRNA transfected (200 MOI) cells. Scale bar: 100 μm.
